# Genomic characterization of *Trichoderma atrobrunneum* (*T. harzianum* species complex) ITEM 908: insight into the genetic endowment of a multi-target biocontrol strain

**DOI:** 10.1186/s12864-018-5049-3

**Published:** 2018-09-11

**Authors:** Francesca Fanelli, Vania Cosma Liuzzi, Antonio Francesco Logrieco, Claudio Altomare

**Affiliations:** Institute of Sciences of Food Production, National Research Council, Bari, Italy

**Keywords:** *Trichoderma*, Comparative genomics, CAZYmes, Biocontrol, Peptaibols, Secondary metabolites, Mycoparasitism, Antagonism

## Abstract

**Background:**

So far, biocontrol agent selection has been performed mainly by time consuming in vitro confrontation tests followed by extensive trials in greenhouse and field. An alternative approach is offered by application of high-throughput techniques, which allow extensive screening and comparison among strains for desired genetic traits. In the genus *Trichoderma*, the past assignments of particular features or strains to one species need to be reconsidered according to the recent taxonomic revisions. Here we present the genome of a biocontrol strain formerly known as *Trichoderma harzianum* ITEM 908, which exhibits both growth promoting capabilities and antagonism against different fungal pathogens, including *Fusarium graminearum*, *Rhizoctonia solani*, and the root-knot nematode *Meloidogyne incognita*. By genomic analysis of ITEM 908 we investigated the occurrence and the relevance of genes associated to biocontrol and stress tolerance, providing a basis for future investigation aiming to unravel the complex relationships between genomic endowment and exhibited activities of this strain.

**Results:**

The MLST analysis of *ITS-TEF1* concatenated datasets reclassified ITEM 908 as *T. atrobrunneum*, a species recently described within the *T. harzianum* species complex and phylogenetically close to *T. afroharzianum* and *T. guizhouense*. Genomic analysis revealed the presence of a broad range of genes encoding for carbohydrate active enzymes (CAZYmes), proteins involved in secondary metabolites production, peptaboils, epidithiodioxopiperazines and siderophores potentially involved in parasitism, saprophytic degradation as well as in biocontrol and antagonistic activities. This abundance is comparable to other *Trichoderma* spp. in the *T. harzianum* species complex, but broader than in other biocontrol species and in the species *T. reesei*, known for its industrial application in cellulase production. Comparative analysis also demonstrated similar genomic organization of major secondary metabolites clusters, as in other *Trichoderma* species.

**Conclusions:**

Reported data provide a contribution to a deeper understanding of the mode of action and identification of activity-specific genetic markers useful for selection and improvement of biocontrol strains. This work will also enlarge the availability of genomic data to perform comparative studies with the aim to correlate phenotypic differences with genetic diversity of *Trichoderma* species.

**Electronic supplementary material:**

The online version of this article (10.1186/s12864-018-5049-3) contains supplementary material, which is available to authorized users.

## Background

The fungal genus *Trichoderma* Pers. comprises numerous species of industrial, biotechnological and agricultural interest [1]. The first comprehensive description of the genus is dated back to 1969 [2] and included nine species-complexes, which grouped morphologically indistinguishable but genetically different species. Recent advancements in *Trichoderma* taxonomy, supported by the molecular analysis of variable regions of genomic DNA with phylogenetic and taxonomic significance, have led to the current system of more than two-hundred different biological species [3]. *Trichoderma* spp. have been known to be antagonistic to plant pathogens since the first half of ‘900 [4], but the interest in antagonistic *Trichoderma* has grown significantly in the last three decades, with the prospect of practical use of these fungi for biological control of plant diseases and as biofertilizers. Intense research on *Trichoderma* has shown that the beneficial effects of some strains go beyond the direct inhibition of plant pathogens by mycoparasitism, antibiosis and competition and include plant growth promotion [5], solubilization of soil micro- and macro-nutrients [6] and activation of plant systemic resistance [7], in a complex three-way interaction amongst antagonist, pathogen and plant.

In recent years, the availability of genomic sequences of a few *Trichoderma* species with different mycoparasitic or ecological behaviors has allowed to investigate the genetic bases of biocontrol, the genes involved and the relevant functions, using a comparative approach [8–10]. The comparative analyses of *Trichoderma* genomes have provided new insights and deeper understanding of the biology of *Trichoderma* species with different lifestyles [8, 11]. Nevertheless, the inference of data provided by genomic analyses with the huge amount of information on *Trichoderma* biology gained by studies conducted over the past years is difficult. Indeed, most of the biocontrol *Trichoderma* species and strains used in past studies were not identified according to current criteria, and most of specimens are no longer available for re-examination. Therefore, our knowledge about biocontrol efficacy, mode of action, metabolite production and other biological functions that in were ascribed to one or to another *Trichoderma* species is jeopardized, and it should be carefully taken and in some cases re-considered. For instance, Chaverri et al. [12] in a revision of the taxonomy of the *T. harzianum* species complex found that none of the strains in four commercial biocontrol products reported as *T. harzianum* were actually *T. harzianum* sensu stricto, but belonged to the new species *T. afroharzianum*, *T. guizhouense* and *T. simmonsii*.

*Trichoderma harzianum* isolate ITEM 908 is a biocontrol strain which exhibits several interesting biological properties. The isolate was originally selected as a biocontrol agent of damping-off and root and stem rots of tomato and other vegetables caused by *Rhizoctonia solani* in heavily infested and “tired” soils. Then ITEM 908 was found capable of promoting growth and development of the root system in tomato and to be rhizosphere competent [13]. ITEM 908 was reported to reduce the inoculum of *Stemphylium vesicarium* and control brown spot of pear after colonization of pear-leaf litter and ground-cover litter in pear orchards [14]. In addition, it was found to antagonize *Fusarium graminearum*, the causal agent of Fusarium head blight of wheat and other grains, and to inhibit the formation and development of peritecia in vitro [15]. Also, it produces metabolites with fagodeterrent activity that are active against aphid pests [16, 17]. Lately it was found that soil pre-treatment with ITEM 908 determined a decrease in the reproduction rate of the root-knot nematode *Meloidogyne incognita* in tomato plants, thus resulting in reduced severity of nematode attack [18]. Also, ITEM 908 was able to modulate the expression of genes associated with plant immune responses and activate plant defense priming against *M. incognita* [19]. The isolate ITEM 908 is currently being registered under the European Union regulation as an active ingredient for the production of commercial biopesticides.

We here present the genome sequence of the *Trichoderma* strain ITEM 908, which we re-classify as *T. atrobrunneum* F.B. Rocha, P. Chaverri & W. Jaklitsch. Also, we performed an analysis of predicted proteins, focusing on those associated to biocontrol mechanisms and risk assessment, such as carbohydrate-active enzymes (CAZYmes), secondary metabolites (SMs), and stress responses. To investigate the relevance of some proteins to the biological functions and lifestyle of ITEM 908, we used a comparative approach [8, 20] based on the annotated gene models of the 20 genome assemblies that were retrievable from the NCBI database at the time we started the analysis (July 2017). The aim of this work was to investigate occurrence and role of some biocontrol-associated genes and provide a basis for future investigations concerned with functions of ITEM 908 and with its interactions.

## Results

### Properties of T. atrobrunneum genome

The genome of ITEM 908 was sequenced using a whole genome shotgun approach on an Ion S5™ platform (Thermo Fischer Scientific) generating around 7 M reads (Table 1), assembled using the Spades v5.0 software for a total of 804 contigs (705 > 1000 bp), a GC% of 49.18 and a mean coverage of 60×. The overall contiguity of the assembly was good, with a N50 of 129 Kbp; the longest assembled fragment was 552 Kbp in length (performed by QUAST, available at http://quast.sourceforge.net/quast) while the total length of the assembly was of 39,131,654 bp.

**Table 1:** Summary of the ITEM 908 genome sequencing and assembly results

Total sequenced bases	2,334,187,798
Mean read length	320 bp
Number of scaffolds	804
Largest contig	552,646
Number reads	7,291,229
N50 reads	129,299
Genome size	39,149,368 bp
GC content	49.18%
Predicted genes	8649

This Whole Genome Shotgun project has been deposited at DDBJ/ENA/GenBank under the accession PNRQ00000000. The version described in this paper is version PNRQ01000000.

### Taxonomic assignment of ITEM 908

**Fig. 1:**
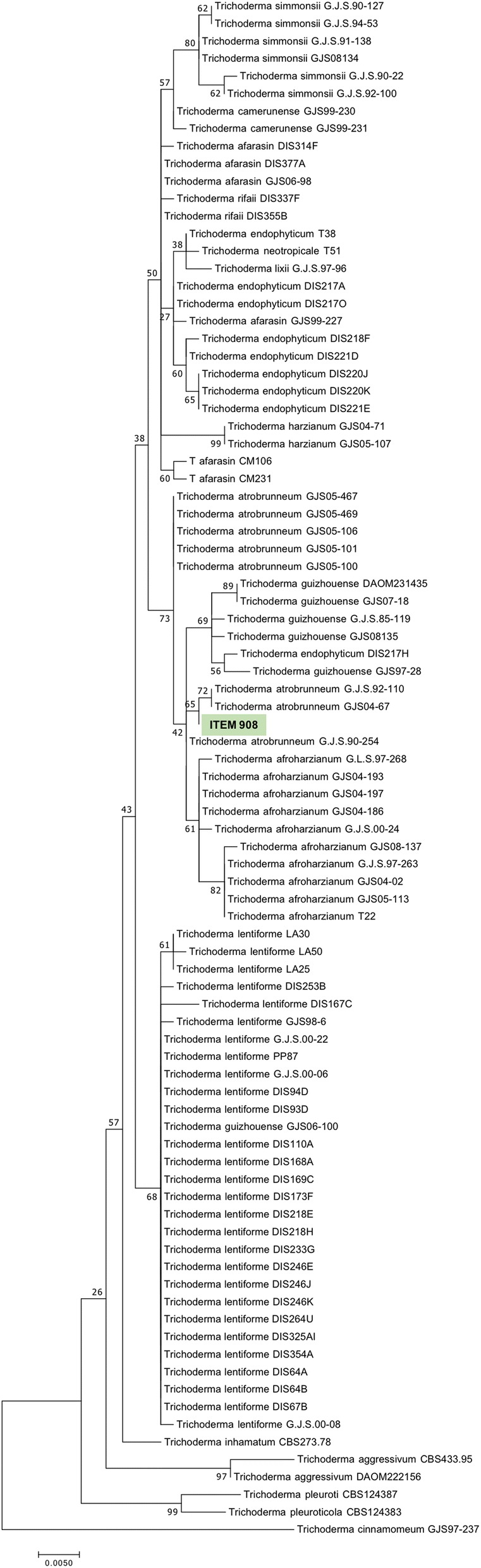
Phylogenetic tree inferred by maximum likelihood analysis (ML) performed on the *ITS-TEF1* concatenated datasets of *Trichoderma* spp. Values at the nodes represent ML bootstrap/BI posterior probability

The Maximum likelihood (ML) analysis performed with all the *ITS*-*TEF1* concatenated datasets sequences of 100 *Trichoderma* spp. isolates (Additional file 1) placed ITEM 908 within the *T. atrobrunneum* group, close to *T. afroaharzianum* and *T. guizhouense* (Fig. 1). Therefore, at the state of the art the strain formerly known as *T. harzianum* ITEM 908 is re-classified as belonging to the species *T. atrobrunneum* F.B. Rocha, P. Chaverri & W. Jaklitsch, a species recently described within the *T. harzianum* species complex and phylogenetically close to *T. afroharzianum* and *T. guizhouense* [12].

### Gene prediction and classification

**Fig. 2:**
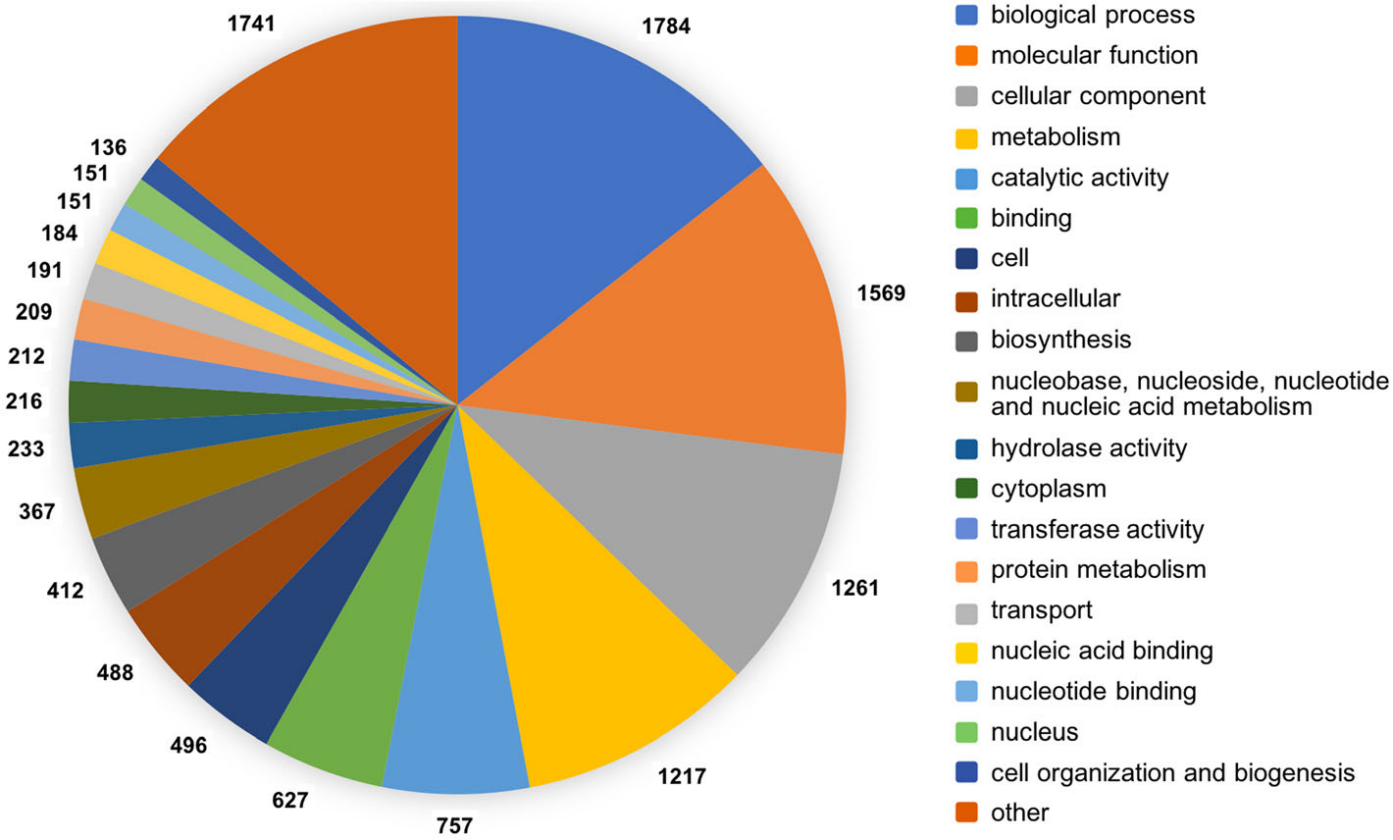
Pie chart representation of GO terms classification count results obtained by CateGOrizer

In the genome of *T. atrobrunneum* ITEM 908 a total of 8649 genes were predicted (Additional files 2 and 3). Among these, the predicted secretome consisted of 761 proteins, comparable with that of close *Trichoderma* species [21]. The analysis of the predicted proteome of ITEM 908 using the PFAM annotator software led to the identification of 12,891 functional pfam domains (Additional file 4). GO terms obtained by pfam mapping were classified using CateGOrizer [22]. One thousand seven hundred and eighty-four GO terms were classified into biological process, 1569 into molecular function and 1261 into cellular component. In particular, 757 domains were classified as catalytic activity, 412 under biosynthesis and 233 under hydrolase activity (Fig. 2).

### Comparative analysis of pfam domains in Trichoderma spp. genomes

In order to gain insight into the endowment of ITEM 908 with genes putatively associated with biocontrol and stress tolerance, 20 genomes of *Trichoderma* spp. retrievable in NCBI database were extracted and genes were predicted using the Augustus v3.1 software; the predicted proteomes of these strains and ITEM 908 were then analyzed using the PFAM annotator software. Among all, 15 pfam domains were selected based on their reported involvement in biocontrol-associated functions, such as stress tolerance and parasitic activities. The results of the comparative analysis are summarized in Table 2.

**Table 2:** Comparison of pfam domains associated to stress tolerance and antagonistic activities in the genomes of *T. atrobrunneum* ITEM 908 and 20 other *Trichoderma* spp. strains with different lifestyles

PFAM domain	Function/Process	*T. atrobrunneum* ITEM 908	*T. asperellum* B05	*T. atroviride* IMI 206040	*T. atroviride* JCM 9410	*T. atroviride* XS2015	*T. gamsii* T6085	*T. guizhouense* NJAU 4742	*T. hamatum* GD12	*T. harzianum* B97	*T. harzianum* T6776	*T. koningii* JCM 1883	*T. longibrachiatum* SMF2	*T. parareesei* CBS 125925	*T. pleuroti* TPhu1	*T. reesei* CBS 999.97	*T. reesei* QM6a	*T. reesei* RUT. C-30	*T. virens* FT-333	*T. virens* Gv29–8	*T. virens* IMI 304061	*T. virens* IMV 00454
ABC trasporter region	Transmembrane transport	60	55	60	60	60	52	68	54	68	61	67	68	67	67	68	68	68	60	60	68	77
Adenylate cyclase	Regulation, light-response	1	1	1	1	1	1	1	1	1	1	1	1	1	1	1	1	1	1	1	1	1
Cellulase glucan 1,3-beta-glucosidase	Synthesis and degradation of complex carbohydrates (CAZyme)	9	8	10	10	10	11	10	10	9	10	8	8	9	9	9	9	8	11	11	15	10
Chitobiosidase	CAZyme	9	10	13	13	13	13	9	12	9	9	8	8	7	7	8	8	8	9	9	10	12
Glutathione transferase	Stress tolerance, detoxification	77	44	65	64	66	62	74	54	66	74	61	60	65	67	64	64	64	66	65	93	83
Glycoside hydrolase	CAZyme	247	220	236	241	235	246	246	228	251	251	212	213	210	232	208	210	206	237	236	288	235
Glycoside hydrolase: Chitinase	CAZyme	24	24	30	31	30	25	23	22	26	27	21	20	22	23	18	19	20	31	31	38	23
Glycoside hydrolase: Xylanase	CAZyme	5	3	5	5	5	5	5	5	5	5	4	4	4	4	4	5	5	3	3	3	6
Hydrophobin	Light-response, adhesion to surfaces	10	14	12	12	10	16	7	17	10	12	11	11	9	10	11	11	11	10	10	12	10
HSP70	Stress tolerance	17	16	18	18	18	17	16	16	18	17	17	18	18	17	17	18	18	17	17	20	19
Kelch motif	Regulation	19	18	19	19	19	19	17	20	20	18	20	20	20	18	20	20	20	18	18	18	51
Methyltransferase	Gene regulation	101	71	89	88	87	87	109	77	102	102	88	88	89	97	89	91	89	89	90	111	112
N-acetyl-beta-D-glucosaminidase	CAZyme	2	2	2	2	2	2	2	2	2	2	2	2	2	2	2	2	2	2	2	2	2
Peptidase	Parasitism	182	139	163	161	162	161	176	145	178	174	154	155	160	173	160	161	162	159	155	182	187
Polygalacturonase	CAZyme	6	4	5	6	5	8	5	6	6	3	3	3	5	4	5	4	3	7	7	6	5

The number of ABC transporters varied within a range of 52 (*T. gamsii* T6085) and 77 (*T. virens* IMV 00454), with 60 pfam domains found in ITEM 908 like in *T. atroviride* IMI 206040, JCM 9410 and XS2015. Considerable variations were found in glutathione transferase (44 to 93 pfam domains) and in the set of peptidases, which ranged from 139 (*T. asperellum* B05) to 187 (*T. virens* IMV 00454); 182 peptidase domains were found in ITEM 908 genome. Only two out of four investigated strains of *T. virens* had the same number (IMI 304061) or more (IMV 00454) peptidases. Also, a comparatively high number of methyltransferase domains were found in ITEM 908. The numbers of HPS70 and kelch motif pfam domains displayed limited variation, ranging from 16 to 20 and from 17 to 20, respectively, with the exception of *T. virens* IMV 00454 that had 51 pfam for kelch motif. One adenylate cyclase pfam domain was retrieved in all the analyzed *Trichoderma* spp. The number of hydrophobin pfam domains ranged from 9 (*T. parareesei* CBS 125925) to 17 (*T. hamatum* GD12). Forty-one phosphopantetheine attachment site pfam domains were found in the genome of *T. atrobrunneum* ITEM 908.

### CAZYmes

*Trichoderma* is a model system for the production of carbohydrate-active enzymes (CAZYmes). This group comprises a list of modules that are classified in the CAZY database (www.cazy.org) as belonging to different families, including glycoside hydrolases (GHs) which catalyze the hydrolysis and/or rearrangement of glycosidic bonds, glycosyl transferases (GTs) that are responsible for the formation of glycosidic bonds, polysaccharide lyases (PLs) which catalyze the non-hydrolytic cleavage of glycosidic bonds, carbohydrate esterases (CEs) which hydrolyze the carbohydrate esters, and auxiliary activities (AAs) which are redox enzymes acting in conjunction with CAZYmes. Due to their important role in parasitism and saprophytic degradation of debris, a focused investigation was performed on the CAZYmes present in the genomes of *Trichoderma* spp. (Additional file 5: Table S1). In the genome of *T. atrobrunneum* ITEM 908, 300 domains were retrieved as associated with CAZYmes, including GH family associated domains, starch binding domains and transferases. The number of CAZYme domains in ITEM 908 genome was the second highest among the analyzed *Trichoderma* species after *T. virens* IMI 304061 where 344 domains were counted, and equal to *T. harzianum* B97 MRYK01.1. In ITEM 908 genome we identified 24 pfam domains of the GH18 family, 17 of which with a chitinase active site as predicted by InterPro [23].

**Fig. 3:**
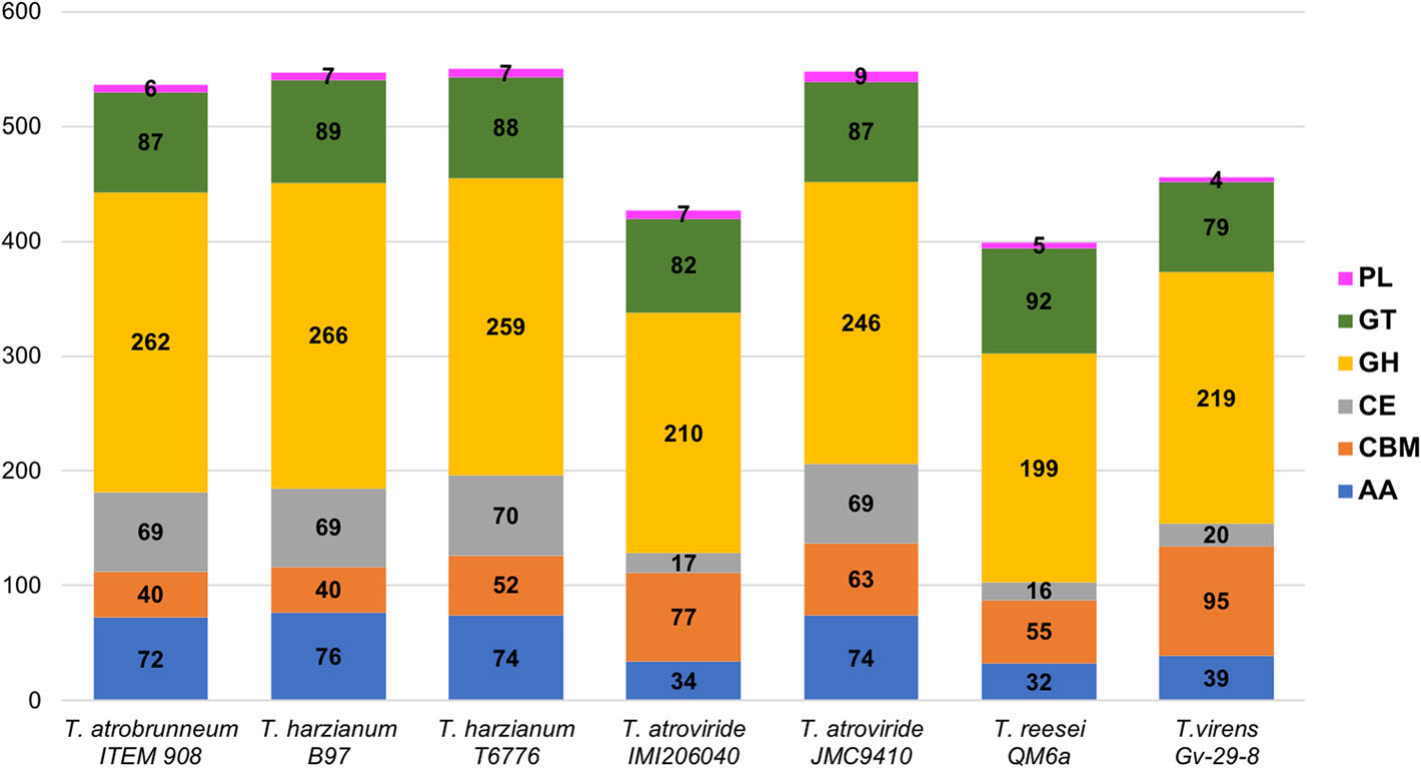
CAZymes harbored by selected *Trichoderma* species. PL = polysaccharide lyases; GT = glycoside transferases; GH = glycoside hydrolases; CE = carbohydrate esterases; CBM = carbohydrate-binding modules; AA = auxiliary activities

**Fig. 4:**
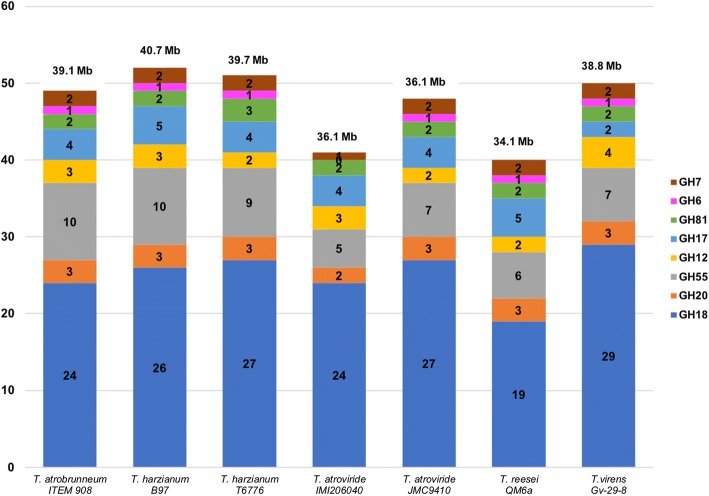
Glycoside hydrolases harbored by selected *Trichoderma* species. GH = glycoside hydrolases; chitinases: GH18 and GH20; glucanases: GH55, GH12, GH17 and GH81; cellulases: GH6 and GH7. Genome size of *Trichoderma* spp. is indicated above the respective bar

The results of the comparative analysis performed by dbCAN (see section Bioinformatic methods) on a restricted number of genomes of *Trichoderma* spp. are shown in Figs. 3 and 4. The genetic endowment of *T. atrobrunneum* ITEM 908 with CAZYmes appears to be similar to that of the representatives of the closely related species *T. harzianum* and significantly higher than *T. atroviride* IMI206040, *T. reesei* QM6a and *T. virens* Gv-29-8 (Fig. 3). *T. atrobrunneum* and *T. harzianum* showed the highest number of GH (around 260 each) compared to other *Trichoderma* species and higher numbers of CE and AA than *T. atroviride* IMI206040, *T. reesei* QM6a and *T. virens* Gv-29-8 (Fig. 3). On the contrary, *T. virens* harbors the highest endowment of Carbohydrate-Binding Modules. Within the GH class of CAZYmes (http://www.cazy.org) a higher number of members of the GH55 family (exo-β-1,3-glucanase and endo-β-1,3-glucanase) was found in *T. atrobrunneum* and in *T. harzianum*, compared to the other analyzed species and isolates (Fig. 4).

**Fig. 5:**
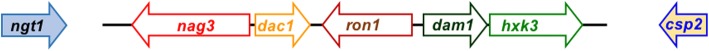
Genomic organization of the GlcNAc gene cluster in *T. atrobrunneum* ITEM 908. Gene clustering is represented by the arrows superposed on the horizontal black line. Intergenic spaces are not drawn in scale

As in all ascomycetes filamentous fungi, in ITEM 908 the genes responsible for the conversion of N-acetyl-glucosamine (GlcNAc) to fructose 6-phospate are clustered. The organization of this cluster in ITEM 908 is identical to that reported for *T. reesei* [24]. The GlcNAc gene cluster (Fig. 5) consists of 5 genes: g4416, coding for a homologue to *hxk3*; g4417, homologue to the glucosamine-6-phosphate deaminase *dam1*; g4418, homologue to the transcription factor *ron1,* (regulator of N -acetylglucosamine catabolism), which belongs to a rare family of exclusively fungal transcription factors; g4419, homologue to the GlcNAc-6-phosphate deacetylase *dac1*; g4420, homologue to β-N-acetylglucosaminidases *nag3*. We also identified three more genes, two of which (g1686, g2395) are homologous to the GlcNAc:H^+^symporter *ngt1*, while the third is an additional transcriptional regulator, homologue of *csp2*. All these three genes are located outside the cluster as in *T. reesei* [24].

### Secondary metabolites (SMs) genes and gene clusters

Like in other well studied fungal species, also in *Trichoderma* spp. the genes for secondary metabolism are included in large biosynthetic clusters comprising core enzymes such as polyketide synthases (PKS), non-ribosomal peptide synthetase (NRPS), terpene synthase/cyclases, and several additional genes including transcription factor, transporters and oxidoreductases, responsible for the biosynthesis of different secondary metabolites through complex enzymatic pathways.

The genome of *T. atrobrunneum* ITEM 908 harbors 18 putative PKS, 8 putative NRPS genes, 5 putative PKS-NRPS, and 5 terpene synthase (TS) genes (Additional file 6: Table S2). These numbers are comparable to what reported for other *Trichoderma* spp., as shown in Table 3.

**Table 3:** Secondary metabolism genes in the genomes of *Trichoderma* spp.

Core genes	*T. atrobrunneum*	*T. reesei* ^a^	*T. atroviride* ^a^	*T. virens* ^a^
NRPS	8	8	9	22
PKS	18	11	15	18
PKS/NRPS	5	2	1	4
TS	5	6	7	11

^a^Zeilinger et al., 2016 [10]

#### Polyketides

In the genome of *T. atrobrunneum* ITEM 908 we identified a genomic locus with similar organization and genic content of the conidial pigment biosynthetic gene cluster described by Atanasova et al. [25] in *T. reesei* and orthologue of pigment-forming PKSs involved in synthesis of aurofusarin and bikaverin in *Fusarium* spp. In *T. atrobrunneum* ITEM 908 the cluster shows the same organization reported for *T. virens* [26] (Additional file 7: Figure S1). In the PKS cluster we also identified the homologue of the uncharacterized protein with oxidoreductase activity TRIVIDRAFT_90482 (g868), the homologue of the endo-chitosanase TRIVIDRAFT_59043 (g869), the homologue of the *Fusarium gip1*, the uncharacterized protein with oxidoreductase activity TRIVIDRAFT_69422 (g870). The uncharacterized protein TRIVIDRAFT_153900, homologue of the *Fusarium aurZ*, was partially predicted as g871, although it misses the initial Met. The gene sequence of the homologue of the *pks4*, the key gene of the cluster (TRIVIDRAFT_209609, the putative conidial pigment polyketide synthase PksP/Alb1 aurPKS), was identified in this genomic location (NODE_11:113238_119919) and the protein was predicted by the EMBOSS© Sixpack sequence translation tool (http://www.ebi.ac.uk/Tools/st/emboss_sixpack/). The homologue of the uncharacterized protein TRIVIDRAFT_49720 was identified as g872, while the homologue of the uncharacterized protein TRIVIDRAFT_192659 was partially predicted as g873.

#### Non-ribosomal peptides

This class of compounds, which display an extremely broad range of biological activities and pharmacological properties, are mainly represented in *Trichoderma* by peptaibols, epidithiodioxopiperazines and siderophores. We analyzed the genome sequence of ITEM 908 for the presence of all the Tex NRPSs described for *T. virens* by Mukherjee et al. [27]. In addition to genes of peptaibol synthetases (*tex1*, *tex2* and *tex3*), we got evidence of the presence of genomic loci coding for the homologues of *tex7* (partially identified as g3551, complete sequence at NODE_16947–36,350), *tex8* (NODE_17:22224–5441), *tex9* (NODE_142:21235–37,370), *tex10* (NODE_7:158284–172,709), *tex16* (Node_26:168533–175,690), *tex19* (NODE_20:103395–97,482), *tex20* (NODE_98:33998–39,293), *tex22* (NODE_26_71758–66,286), *tex23* (NODE_142:20622–17,120), *tex24* (NODE_26:151273–148,142), *tex25* (NODE_140:25454–26,758) and *tex26* (NODE_26:164315–165,994) [27]. As reported for *T. virens*, the genes coding for Tex16, Tex24 and Tex26 are located in a putative gene cluster.

##### Peptaboils

The genome of ITEM 908 harbors three genomic loci with sequences potentially coding for homologues of peptaibol synthetases described in *T. virens* by Mukherjee et al. [27, 28]. These proteins are characterized by a multimodular architecture [29] and are referred as Tex1 (TRVIDRAFT_66940), Tex2 (TRIVIDRAFT_10003) and Tex3 (TRIVIDRAFT_69362). On the basis of sequence alignment, 3 genomic loci were identified: one for tex1 in the NODE_14:201111–263,835, one for Tex2 in the NODE_29:76842–26,784 and one for Tex3 in the NODE_36:89140–64,378.

##### Epidithiodioxopiperazines (ETPs)

**Fig. 6:**
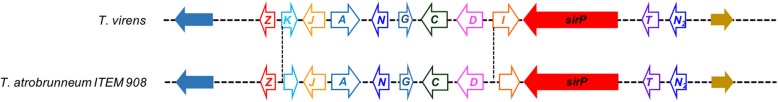
The SirP cluster in *T. virens* and *T. atrobrunneum* ITEM 908. The red arrow indicates the non-ribosomal peptide synthetase sirP. Z: zinc finger transcriptional regulator; K: G-Glutamyl cyclotransferase; J: dipeptidase; A: MFS transporter; N: methyltransferase; G: glutathione transferase, C: cytochrome P450 monooxygenase; D: dimethylallyl transferase; I: aminotransferase; T: thioredoxin reductase; N2: methyltransferase

In *Trichoderma*, the presence of gene clusters responsible for the biosynthesis of the ETPs sirodesmin (SirP cluster) and gliotoxin (GliP cluster, 12 genes) has been reported [8, 30]. In *T. atrobrunneum* ITEM 908 we did not find the GliP cluster, similarly to *T. atroviride* [30], but we identified a genomic locus homologous to the SirP cluster comprised in the NODE_77:109590–134,547. The gene cluster has the same content and organization as the cluster in *T. virens* [31], with the exception of the aminotransferase *sirI* and the G-Glutamylcyclotransferase *sirG*, in which the predicted proteins missed the first 16 and 17 aminoacids, respectively (Fig. 6).

##### Siderophores

In *Trichoderma* spp. three NRPSs responsible for siderophore biosynthesis are located in three different gene clusters [10]. The first cluster is responsible for the production of the intracellular ferricrocin. In the genome of ITEM 908 we identified the homologues of the aldehyde dehydrogenase (g626), the oxidoreductase (g625), the NRPS (TRIVIDRAFT_85582 = tex10) (g624), the orntithine monooxygenase (g623) and the transcription factor (g622) (Additional file 8: Figure S2).

The second gene cluster comprise NPS6, a key enzyme which is responsible for extracellular siderophore production in *T. virens* (ID 44273, [20]) and also found in *T. atroviride* (ID 39887) and *T. reesei* (ID 67189). In ITEM 908 the orthologue of this protein was predicted as g4381, with 89% identity with ID 44273 (genomic locus NODE_98:33998–39,283). We also identified the other proteins comprised in the cluster, g4380 orthologue of the AMP-binding protein (96% identity with TRIVIDRAFT_44194), g4379 orthologue of the acyl CoA acyltransferase (94% identity with TRIVIDRAFT_44039), g4378 orthologue of the MFS transporter (90% identity with TRIVIDRAFT_43838), g4377 orthologue of the oxidoreductase (90% identity with TRIVIDRAFT_44141) and g4376, orthologue of the ABC transporter (94% identity with TRIVIDRAFT_210053).

There is an additional genomic locus which comprised a hypothetical cluster with a NRPS siderophore synthase orthologue to the *Aspergillus fumigatus sidD* gene (or *sid4* in *Neurospora crassa*) (Tr_71005 o Tv_70206 = TRIVIDRAFT_192365). In ITEM 908 we identified a similar locus which contains gene sequences coding for homologues of the NRPS (Tr_71005/ITEM_908_NODE_171:59024–64,759; Tv_70206/ITEM_908_NODE_171:58575–64,402), the ABC transporter, the Acyl-CoA acyltransferase, one hydrolase and one protein with ClpP/crotonase-like domain. While this genomic content is conserved and shared by *T. reesei*, *T. virens* and *T. atrobrunneum*, the gene flanking the NRPS is annotated as an uncharacterized short-chain dehydrogenases/reductases (SDR) family in *T. virens* (TRIVIDRAFT_51816 fungi.ensamble.org) and as an ABC transporter in *T. reesei* (sid6 transacylase TRIREDRAFT_82626). In ITEM 908 these gene orthologues are located in two different genomic contents, predicted as NODE_1_length_552646_cov_38.83.g10 (identity of 86%) and as NODE_9_length_343799_cov_42.23.g747 (identity of 83%) respectively [32].

#### Terpenoids

Trichothecenes are sesquiterpenoid epoxides that are formed starting from the parent compound trichodiene through isomerisation–cyclisation of farnesyl pyrophosphate. This reaction is catalysed by the key enzyme trichodiene synthase, encoded by tri5 gene. *Tri5* and other genes of trichothecene biosynthesis are organized in a coordinately regulated gene cluster. In ITEM 908 genome we identified a partial domain similar to *tri5* (identity< 30%). The identified gene is not complete, and there is no evidence of the presence of other trichothecene cluster genes in the same genomic location. Similarly, there is no evidence of the presence of the biosynthetic cluster for viridin, a fungistatic and anticancer compound produced by both ‘P’ and ‘Q’ strains of *T. virens* [33].

## Discussion

A comparatively high number of peptidases and methyltransferase domains were found in ITEM 908. Methyltransferases are implicated in regulation of gene expression, and in *Trichoderma* they have been found to be associated to CAZYmes synthesis and to developmental and trophic functions, such as sporulation and mycoparasitism [34, 35]. In *T. reesei*, CAZYme encoding genes including those of cellulases, hemicellulases and chitinases, are located in clusters [36], and this organization has also been found in ITEM 908 genome. Marie-Nelly et al. [37] noted that some of the CAZYme clusters in *T. reesei* were located in the subtelomeric regions, close to the chromosomal ends. In addition, in *T. reesei* regions containing CAZYme gene clusters were reported to contain also clusters of genes of SM biosynthesis, such as NRPS and PKS, and their distribution is not random, since they are colocalized in discrete regions [36]. This localization is consistent to what reported for SM in other phylogenetically distant fungi [38] and suggest the possibility to rapidly generate genetic diversity which confers an advantage for colonization and antagonistic activity. The co-localization of CAZYme and SM genes in the same regions suggests a possible co-regulation of these genes, confirming the biological importance of CAZYmes clustering. In *Aspergillus* and *Fusarium*, clusters of SM genes have been found to be regulated at the level of histones by the enzyme methyltransferase LaeA [39–42], which also regulates important functions such as conidiation and development, thus affecting the fitness and adaption of the fungus to the environment [43]. LAE1, the orthologue of LaeA, was recently identified in *T. atroviride* and *T. reesei* genomes [34, 35], where it is essential for asexual development and mycoparasitism, modulating the production of both CAZYmes and SM. In *T. atrobrunneum* ITEM 908 the putative orthologue of LAE1 was predicted as g8186, with an identity of 74% and 69% with the proteins identified in *T. reesei* and *T. atroviride* respectively. The identity reached 98% with the hypothetical protein THAR02_09616 (GenBank accession KKO98277.1) of *T. harzianum* strain T6776. The protein was functionally classified as a S-adenosyl-L-methionine-dependent methyltransferase by InterPro [23]. Given the role of the methyltransferase in regulation and co-regulation of CAZYmes and SM biosynthesis, the large number of methyltransferases retrieved in ITEM 908 suggest that also in this strain biocontrol-associated metabolism and biosynthetic activities may be subjected to strong epigenetic regulation. In this regard, further investigations on the role of methyltransferases in regulation and fine tuning of gene expression as for biocontrol- and ecological fitness-associated functions in *Trichoderma* appear of utmost interest.

Peptidases and proteases catalyze the cleavage of peptide bonds within proteins and are involved in a broad range of biological processes in all organisms. Since fugal cell walls contain lipids and proteins beside chitin and glucan, the involvement of peptidases in mycoparasitism has been hypothesized [44–51]. The basic proteinase-encoding gene *prb1* was found in the genome of the plant cell wall degrader *T. reesei*, as well as in other *Trichoderma* spp. and, in disagreement with what reported by Herrera-Estrella and coworkers in 1993 [52] based on hybridization techniques, the protein appears to be highly conserved among *Trichiderma* spp. In *T. atrobrunneum* ITEM 908 the predicted proteinase g1426 displays 93% of identity with *T. atroviride* (XP_013940434.1) and *T. harzianum* (AAA34211.1), 92% with *T. virens* Gv29–8 (XP_013954377.1), 92% with *T. viride* (GenBank accession AIZ77170.1) and 91% with *T. reesei* (XP_006964613.1). The overexpression of *prb1* gene in *T. harzianum* was proven to enhance the biocontrol activity [46] and the presence of this protease in other species may be exploited to improve biocontrol efficacy also in other strains.

Proteases are interesting products of *Trichoderma* not only because of their possible contribution in fungal cell wall degradation during mycoparasitism, but also for their putative involvement in the interaction with a number of different organisms in different ways. Viterbo and co-workers [53] identified two extracellular aspartyl proteases, namely PapA and PapB, which were induced in *T. asperellum* during colonization of cucumber roots and were suggested to have a role in establishment of the plant-*Trichoderma* symbiosis. PapA of *T. asperellum* had 58% similarity to PapA from *T. harzianum*, and the encoding gene *papA* was found to be upregulated by the challenging of *Rhizoctonia solani* in confrontation tests [53]. In *T. atrobrunneum* ITEM 908 BLASTP analysis retrieved homologues of proteases encoded by *papA* and *papB* with 68% and 85% of identity respectively (g3974 and g1149).

An 18-kD protein from *T. virens*, which displayed at the amino-terminal a high similarity to a fragment of serine protease from *Fusarium sporotrichioides*, was identified as an elicitor of plant defense responses [54]. The conserved pfam domain included in this protein is related to the cerato-platanin family containing a number of fungal cerato-platanin phytotoxic proteins approximately 150 residues long. The orthologue of this protein in *T. atrobrunneum* (g2233) displayed 79% of identity with *T. virens* although about 30 aa shorter. Furthermore, in *T. atrobrunneum* genome we counted 5 occurrences of the pfam domain of cerato-platanin in three different proteins.

Proteases also act as proteolytic inactivators of pathogen enzymes and virulence factors [55]. Proteases play also an important role in the mode of action of entomopathogenic [22, 56] and nematophagous [57, 58] microorganisms. Generally speaking, it seems that production of proteinases may play an important role in most pathogen/host interactions. In ascomycetes, plant or insect pathogens were reported to retain peptidases that were mostly lost in saprophytic lineages [22]. In *T. harzianum*, a trypsin-like acidic serine peptidase encoded by *pra1* and the previously cited alkaline serine peptidase PRB1, were reported to have nematicidal activity [49, 59]. Szabó et al. [60] showed that the acidic serine protease pra1, aspartic proteases p6281, the metalloendopeptidase p7455 and the sedolisin serine protease p5216 were co-expressed by *T. harzianum* during parasitization of eggs of the nematode *Caenorhabditis elegans* and suggest a major role of these enzymes in the process. In ITEM 908 we found a PRA1 with 99% identity with PRA1 from *T. harzianum.*

In ITEM 908, the arsenal of peptidases domains appears to be significantly larger than in most of the species and strains examined. Despite the potential relevance of the proteolytic activity for *Trichoderma* biocontrol properties, the number of protease genes cloned to date is relatively low compared with those of other biocontrol-associated enzymes. Although in closely related species data on the co-expression of protease encoding genes might imply a common regulatory network, in our strain further studies are needed to determine how many and which genes are associated to the biocontrol capabilities against plant pathogens, insects and nematodes exhibited by this strain.

The glutathione transferases (GSTs, also known as glutathione S-transferase) represent an important group of (mainly) cytosolic enzymes which detoxify both endogenous and exogenous toxic compounds. Relatively little is known about GSTs from fungi and a very limited number of genes have been cloned and sequenced, so far [61]. Dixit and co-workers [62] succeeded in enhancing the Cadmium tolerance of tobacco plants by heterologous expression of a GTS from *T. virens*. The genetically modified plants showed lower lipid peroxidation and higher levels of antioxidant enzymes, such as GTS, superoxide dismutase, ascorbate peroxidase, guaiacol peroxidase and catalase. In a subsequent work [63], the same research group cloned a GTS encoding gene from *T. virens* and expressed it in transgenic tobacco plants. Transgenic plants proved to be able to degrade the xenobiotic compound anthracene, while no degradation was observed in wild-type plants. Also, in *T. reesei*, GTS seem to have a role in degradation of lignocellulosic biomass [64]. A comparatively high number of GTS pfam domains (77) were found in the genome of ITEM 908, which is presumptively indicative of a high number of GTS encoding genes. Enzymes of the GTS family have an outstanding potential for biotechnological applications. They may play a role in various functions associated to biological control and bioremediation, including tolerance to fungicide and agrochemicals, detoxification of toxins produced by competing microorganisms, mitigation of oxidative stress, degradation of recalcitrant polyaromatic pollutants and breakdown of lignocellulosic materials. Also, the possibility exists of heterologous expression of these enzymes in transgenic plants to enhance tolerance to biotic and abiotic stresses. The investigation of catalytic and structural diversity of GSTs of ITEM 908 may open interesting prospects of successful applications in the above fields of research and biotechnology.

ABC transporters are transmembrane proteins that have the ability to transport molecules, such as toxins, ions and proteins into and out of cells by an energy-consuming process involving ATP hydrolysis. They are known to contribute to resistance against toxic compounds in microbial pathogens and tumor cells. In *Trichoderma*, ABC transporters are thought be involved in extrusion of endogenous metabolites and protection from exogenous toxicants, such as plant phytoalexins, microbial toxins and pesticides [65]. Schmoll et al. [9] found a strong expansion in the number of ABC transporters in *T. virens, T. atroviride*, and *T. reesei* compared to *N. crassa*, *S. cerevisiae*, or *S. pombe* and suggested that this reflects the adaption to their ecological niches and lifestyles, i.e., mycoparasitism competition with other soil organisms and plant litter decomposer. We identified 60 ABC transporters in ITEM 908 and 52 to 77 in the others 20 isolates examined, which is consistent with the findings of Schmoll et al. [9].

CAZYmes in *Trichoderma* spp. play a central role in both mycoparasitism and degradation of plant residues. For this reason, they are regarded as indicators of bio- or necrotrophic parasitic lifestyle, and of saprophytic capability of colonizing plant litter [8, 9]. The main constituents of the fungal cell wall are chitin and glucan. Conversely, in plant cell wall the major carbohydrates are cellulose, hemicelluloses and pectins. According to Schmoll et al. [9], the ecological behavior of the mycoparasites *T. atroviride* and *T. virens*, compared to the plant wall degrader *T. reesei*, is reflected by the sizes of the respective genomes. The smaller size of T*. reesei* genome (34.1 Mbp versus 36.1 and 38.8 Mbp of *T. atroviride* and *T. virens*) is conceivably due to the loss of gene functional to mycoparasitism during the evolution of *T. reesei* [8]. In the genome of ITEM 908 (39.2 Mbp) we found the highest number of CAZYme domains among the species and the strains analyzed except for *T. virens* IMI 304061, with a significantly higher relative abundance of enzymes in the GH (glycoside hydrolases), CE (carbohydrate esterases) and AA (auxiliary activity) families and a smaller proportion of non-catalytic carbohydrate-binding modules (CBM). Among the carbohydrate hydrolases, the family GH18, containing enzymes involved in chitin degradation, is strongly represented in *Trichoderma*, particularly in *T. atroviride* and *T. virens* that have been reported to contain the highest number of chitinolytic enzymes of all described fungi [8]. Since chitin is the main component of fungal cell walls, secretion of chitinolytic enzymes is essential for mycoparasitization and also part of the combined synergistic action with peptaibol antibiotics that leads to prey death [66]. In *T. atrobrunneum* ITEM 908 genome we identified 24 domains of the GH18 family, comparable to the other species in the *T. harzianum* species complex *T. guizhouense* and *T. harzianum*. Glucanases are inductively produced by mycoparasitic *Trichoderma* species grown on media containing chitin or fungal cell walls as the sole carbon source [67, 68] and also have been reported to be induced during mycoparasitism [69]. Comparative genome analysis revealed that the mycoparasites have more glucanases than *T. reesei* [9]. Consistently with the findings of Schmoll et al. [9], the number of cellulases and xylanases found in ITEM 908 was not significantly higher than in other *Trichoderma* spp. genomes, supporting their conclusion that a low variation in cellulases and xylanases is a common feature of the genus *Trichoderma*.

Despite the high number of PKS genes identified in *Trichoderma* spp., the related biosynthetic pathways are not yet characterized. In fungi, PKS are often associated with synthesis of pigments. In *T. reesei* Atanasova et al. [25] identified the gene *pks4* as an orthologue of pigment-forming PKS involved in synthesis of aurofusarin and bikaverin in *Fusarium* spp. Deletion of the key gene *pks4* affected conidia pigmentation and cell wall stability, secondary metabolites production and antagonistic activity of *T. reesei* [25]. Recently, two PKS genes, *pksT-1* and *pksT-2,* were isolated from *T. harzianum* and found to be differentially expressed during challenge with the plant pathogenic fungi *Rhizoctonia solani*, *S. sclerotiorum* and *F. oxysporum* [70]. We identified a gene similar to *pks4* in the genome of *T. atrobrunneum* ITEM 908, but its relevance for biocontrol ability and physiology of ITEM 908 remains to be ascertained.

The analysis made by Mukherjee et al. [27] on the genome of *T. virens* Gv29–8 is so far the most extensive study on the occurrence and role of NPKS and hybrid NRPS/PKS in *Trichoderma*. Based on similarity of sequences and knock-down experiments, the authors predicted putative functions for some of the 22 NPKSs (Tex1–10 and 15–26) and four hybrid NRPS/PKS (Tex11–14) identified. In ITEM 908 we found homologues of Tex1–3 (peptaibol synthetase), Tex10 (ferrichrome synthetase), Tex20 (siderophore synthetases) and Tex19 (sirodesmin synthetase). We also found orthologues of the genes *tex2*, *tex7–8*, and *tex25* that in *T. virens* were upregulated in the presence of maize roots [27] and that are therefore conceivably involved in the plant-fungus interaction, although in ways that are still unknown.

Peptaibols are linear or, in few instances, cyclic peptides made of 4–21 residues and characterized by the presence of the nonproteinogenic amino acid α-aminoisobutyric acid and, in some cases, of isovaline. Peptaibols have plasma membrane-permeabilizing properties and have been associated to important biological functions in *Trichoderma*, such as mycoparasitism, for which they operate synergistically with secreted hydrolytic enzymes [71] and elicitation of plant defense response [72]. Production of peptaibols is widely diffused in species of *Trichoderma* [73] that can synthesize a number of different forms, often showing microheterogeneity [74]. This is due to the ability of single peptaibol synthetases to produce a variety of peptaibols by a module skipping mechanism [27, 75]. So far three genes of paptaibol synthetases, named *tex1*, *tex2* and *tex3* have been identified in *T. virens* genome. Tex1 is a long-chain peptide (18–25 residues) peptaibol synthetase, and it is involved in the production of 18-residue peptaibols [29]. The 18-residues product of Tex1 was proven to be an elicitor of systemic resistance [72]. Later, the short peptaibol synthetase gene t*ex2*, encoding a 14-module enzyme able to assemble both 11-residue and 14-residue peptaibols, was characterized [28]. The third peptaibol synthetase gene (*tex3*), homologous to *tex1* has seven complete modules arranged in a linear fashion [27]. We found homologues of all of these three genes in the genome of *T. atrobrunneum* ITEM 908.

ETPs are molecules with toxic activity conferred by the capability to generate reactive oxygen species by cross-linking proteins via the ETPs disulphide bridge [76]. The ETP gliotoxin has antifungal activity and so far, it has been found only in “Q” strains of the species *T. virens* and its role in antagonism to *Rhizoctonia* has been recognized. Gliotoxin is absent in “P” strains of *T. virens* that instead produce the ETP gliovirin, which has potent antimicrobial properties particularly against *Pythium* and other oomycetes. The production of ETPs is discontinuous in the fungal kingdom. The gene clusters responsible for the production of the ETPs sirodesmin (SirP cluster) and gliotoxin (GliP cluster, 12 genes), were first characterized in the ascomycetes *Leptosphaeria maculans* and the human pathogen *Aspergillus fumigatus* [77, 78], respectively, but then identified also in *Trichoderma* spp. [8, 30]. SirP and GliP clusters of *A. fumigatus* and *L. maculans* are 55 and 28 kb in length, respectively and share ten genes. The GliP cluster present in *T. virens* genome consists of 8 genes only, but its association with gliotoxin biosynthesis has been proven [79]. A GliP cluster is present in *T. reesei*, even though this species does not produce gliotoxin [30]. *Trichoderma virens* also has a gene cluster similar to SirP, encoding a so far unidentified secondary metabolite [30]. However, since none of the SirP gene cluster members has been found to be expressed in *Trichoderma* during mycoparasitism (C. P. Kubicek, unpublished results cited in [30]), the cluster might be not functional, or the metabolite might be not required for antagonism. In *T. atrobrunneum* ITEM 908 we identified only a genomic locus homologue to the SirP cluster and did not find the GliP cluster. Since gliotoxin has potential non-target toxic effects [76], the absence of GliP cluster and the resulting inability to produce gliotoxin is of importance for risk assessment and authorization of ITEM 908-based plant protection products.

Siderophores are small metal-chelating molecules produced by several microorganisms under low iron conditions to chelate the ferric iron [Fe(III)] from the surrounding environment. They also form complexes with other essential micro elements and make them available to the plant [80]. Siderophores play a role in biocontrol of plant diseases by causing Fe starvation of phytopathogens, thus fostering successful competition by biocontrol agents [81]. Also, microbial siderophores play a major role in fertility of soils, plant health and plant nutrition [82]. Fungal siderophores are comprised in three main groups: fusarinines, coprogens and ferrichromes, all belonging to the hydroxamate-class [83]. On average, *Trichoderma* spp. are able to produce 12–14 siderophores [84]. *Trichoderma* siderophores are regarded as part of the mechanism of biocontrol [85] and plant growth promotion [86]. As reviewed by Zeilinger et al. [10], in *Trichoderma* spp. the NRPSs responsible for siderophore biosynthesis are located in three different gene clusters. Accordingly, in ITEM 908 genome we found three NRPS homologues of the NRPS responsible for the synthesis of ferricrocin, of NPS6 involved in synthesis of extracellular siderophores, and of the siderophore synthase *sidD*, which were arranged in three distinct clusters. Ferricrocin is responsible for intracellular storage of iron and is involved in protection of cells from oxidative stress [87]. The extracellular siderophore produced by NPS6 also contributes to protection of the fungus from oxidative stress [88]. Previous studies on NRPSs involved in siderophore biosynthesis in *Trichoderma* reported that the genomes of *T. reesei*, *T. virens* and *T. atroviride* all have a single gene for ferricrocin synthesis; genes orthologues of NPS6 and SidD are in the genomes of *T. reesei* and *T virens*, while *T. atroviride* harbors only the NPS6 orthologue [8, 30].

Trichothecenes represent a large family of terpenoid mycotoxins produced by a variety of filamentous fungi, and most notably by species belonging to the genera *Fusarium*, *Myrothecium* and *Stachybotrys* [89]. The trichothecenes trichodermin, its deacetyl derivative trichodermol and harzianum A have been identified in cultures of *Trichoderma* spp. [90]. The *tri5* gene encoding for trichodiene synthase, the key enzyme of trichothecene biosynthesis was first characterized in *Trichoderma* by Gallo et al. [91]. Unlike in *Fusarium*, in *Trichoderma* this gene is located outside the trichothecenes biosynthetic cluster (TRI) [92]. So far, the only well-characterized *Trichoderma* strains that were reported to harbor orthologues genes of the *Fusarium* TRI cluster belong to the species *T. arundinaceum* and *T. brevicompactum* [92]. Similarly to gliotoxin, the ability to produce trichothecenes can be a safety issue as for registration of *Trichoderma*-based biopesticides. In this regard, the genome of *T. atrobrunneum* ITEM 908 does not harbor either the TRI gene cluster or the *tri5* gene and this rules out the risk of occurrence of trichothecenes in formulations and treated plants.

## Conclusions

For decades biocontrol strains of *Trichoderma* have been selected through in vitro confrontation tests followed by extensive trials in the greenhouse and the field. This trial and error procedure, besides being time and labor consuming, has often led to misleading conclusions and unreliable biocontrol. A drastic change of prospect has come with science-based improvement of biocontrol performances based on understanding of the mechanisms of action and the complex plant-pathogen-biocontrol agent interactions. Molecular techniques have allowed more in-depth studies on this subject, but the interpretation of the results has been in some extent limited by the lack of genome sequence information for biocontrol *Trichoderma* species or strains [93]. In this regard, the presentation of the genome of the multi-target biocontrol strain ITEM 908, belonging to the newly constituted species *T. atrobrunneum*, is a contribution to the understanding of the mode of action and the identification of activity-specific genetic markers that can be used for selection and improvement of biocontrol strains. It extends the number of genomes available for comparative studies aiming to correlate phenotypic differences with genetic diversity of *Trichoderma* species. In conclusion, this work provides a basis to further “omics” studies for a deeper understanding of the complex interactions of this strain with its multiple targets and with plants, and for the development of a knowledge-driven selection of effective *Trichoderma* biocontrol strains.

## Methods

### Genome sequencing and assembly

The antagonistic strain *T. atrobrunneum* (formerly *T. harzianum*) ITEM 908 was originally isolated from soil collected in Apulia, Italy, and maintained in the Agri-Food Toxigenic Fungi Culture Collection of the Institute of Sciences of Food Production, CNR, Bari (http://www.ispa.cnr.it/Collection). A monoconidial culture was grown for 5 days on PDA (Potato Dextrose agar, Oxoid, Italy). Fungal mycelium was then scraped off the agar surface and ground in a mortar using liquid nitrogen to a fine powder. DNA extraction was performed from 10 mg of lyophilized material using the “Wizard ® Magnetic DNA purification system for Food” (Promega, Madison, WI, USA), according to the manufacturer’s instructions. The quantity and quality of isolated DNA was determined a NanoDrop-2000 (Thermo Fisher Scientific, Wilmington, DE, USA) and a Qubit 3.0 fluorometer (Life Technologies). DNA was then subjected to whole genome shotgun sequencing using the Ion S5™ library preparation workflow (Thermo Fisher Scientific, Waltman, MA, USA). Four hundred bp mate-paired reads were generated on the Ion S5™ System (Thermo Fisher Scientific). Duplicate reads were removed by FilterDuplicates (v5.0.0.0) Ionplugin. De novo assembly was performed by AssemblerSpades (v.5.0) Ionplugin™.

### Phylogenetic analysis

Species assignment of ITEM 908 was achieved by application of the genealogical concordance phylogenetic species recognition concept based on the gene sequences of the internal transcribed spacer (*ITS*) and translation elongation factor 1-α (*TEF1*), recognized as the most informative for species discrimination in *Trichoderma* [12]. Genomic sequence of *ITS* and *TEF1* were extracted from ITEM 908 assembly, using the corresponding loci sequences of *T. harzianum* strain T22 (NCBI Accession n. KX632495.1 and KX632609.1) as query. To construct the phylogenetic tree, we used the *ITS/TEF1* concatenated datasets of a total of 100 *ITS1-IT2* and *TEF1 Trichoderma* spp. sequences retrieved by GenBank (https://www.ncbi.nlm.nih.gov/genbank/). Molecular phylogenetic analysis was performed by Maximum Likelihood (ML) method. Consensus tree was inferred using the neighbour-joining method using MEGA v7.0.18 (http://www.megasoftware.net/). Phylogenetic robustness was inferred from 1000 replications to obtain the confidence value for the aligned sequence dataset.

### Bioinformatic methods

Genes were predicted using the Augustus v3.1 software implemented in the Galaxy platform [94] with a model trained on *T. reesei* [32] (gene annotation and mapping available at http://trichocode.com/index.php/t-reesei). The predicted proteins were submitted to the PFAM annotator tool within the Galaxy platform (Galaxy Tool Version 1.0.0) in order to predict the pfam domains.

To analyze the Gene Ontology (GO) terms for all the pfam domains predicted we mapped them against the list generated from data supplied by InterPro for the InterPro2GO mapping [95, 96]. The web-based program Categorizer [97] was used to analyze and classify the GO terms for all the identified domains.

The secretome of *T. atrobrunneum* ITEM 908 was in silico predicted by SignalP [98] (http://www.cbs.dtu.dk/services/SignalP/). This software predicts the presence of signal peptide cleavage sites at the N-terminus in amino acid sequences of a protein, which are used to move it into the endoplasmic reticulum and for secretion.

To perform the comparative analysis of selected pfam domain we retrieved the genomes of 20 *Trichoderma* strains available in GenBank (Additional file 9: Table S3). For each genome genes were predicted using Augustus v3.1 software and pfam domains annotated using the PFAM annotator tool implemented in the Galaxy platform. Fifteen pfam domains (Table 2) were selected based on their involvement in stress tolerance and antagonistic activities.

The CAZYmes annotation was performed by dbCAN (release 6.0, available at http://csbl.bmb.uga.edu/dbCAN/; [99]), web server and DataBase for automated Carbohydrate-active enzyme Annotation.

For the analysis of secondary metabolites genes and gene clusters, enzyme sequences were downloaded from the genome site of *T. virens Gv29–8* (http://genome.jgi-psf.org/TriviGv29_8_2/ TriviGv29_8_2.home.html), *T. atroviride* (http://genome.jgi-psf.org/Triat2/Triat2.home.html) and *T. reesei* (http://genome.jgi-psf.org/Trire2/ Trire2.home.html). The homology-based relationship of *T. atrobrunneum* ITEM 908 predicted proteins towards selected proteins was determined by BLASTP algorithm on the NCBI site (http://blast.ncbi.nlm.nih.gov/Blast.cgi). Gene models were determined manually, and clustering and orientation were subsequently deduced for the closely linked genes.

## Additional files

Additional file 1: The file contains the multi sequence alignment of *ITS-TEF1* concatenated datasets used for the phylogenetic analysis of *Trichoderma* spp. (MAS 69 kb)

Additional file 2: *Trichoderma atrobrunneum* ITEM 908 protein sequences: the file contains the multifasta of the proteins predicted by Augustus [100] implemented in the Galaxy platform (Galaxy tool Version 1.0.0) (FASTA 5102 kb)

Additional file 3: The file contains gene coordinates of *Trichoderma atrobrunneum* ITEM 908. (GTF 26492 kb)

Additional file 4: The file contains the PFAM functional domains of *Trichoderma atrobrunneum* ITEM 908 predicted by the PFAM annotator tool implemented in the Galaxy platform (Galaxy tool Version 1.0.0). (TXT 1927 kb)

Additional file 5: **Table S1.** Comparative analysis of PFAM domains associated to CAZYmes in *Trichoderma* spp. (XLSX 42 kb)

Additional file 6: **Table S2.** Terpene synthase (TS), non-ribosomal peptide synthetase (NRPS), polyketide synthase (PKS) and hybrid PKS-NRPS genes identified in the genome of *T. atrobrunneum* ITEM 908, predicted protein sequences, their putative orthologue and % of identity in *Trichoderma* spp. (XLSX 54 kb)

Additional file 7: **Figure S1.** The putative conidial pigment PKS gene clusters of *Trichoderma spp*. Numbers over the arrows in *T. virens* and *T. reesei* indicated the ID of genes as reported in Ensembl Fungi©. Numbers over the arrows in *T. atrobrunneum* indicated the ID of genes as predicted by Augustus [100]. (PNG 844 kb)

Additional file 8: **Figure S2.** Ferricrocin gene cluster in *T. atrobrunneum* ITEM 908. ALDH: aldehyde dehydrogenase; OXD: oxidoreductase; NRPS: non-ribosomal peptide synthetase; OMO: ornithine monooxygenase; TF: transcription factor. (PNG 142 kb)

Additional file 9: **Table S3.** *Trichoderma* spp. and genomic sequences accession used in this study. (DOCX 13 kb)

## Abbreviations

AA: Auxiliary activity
ALDH: Aldehyde dehydrogenase
CAZYmes: Carbohydrate active enzymes
CBM: Non-catalytic carbohydrate-binding modules
CE: Carbohydrate esterase
ETP: Epidithiodioxopiperazines
GH: Glycoside hydrolase
GlcNAc: N-acetyl-glucosamine
GO: Gene Ontology
GST: Glutathione transferase
GT: Glycosyl transferase
ITS: Internal transcribed spacer
ML: Maximum likelihood
NRPS: Non-ribosomal peptide synthetase
OMO: Ornithine monooxygenase
OXD: Oxidoreductase
PK: Polyketide synthase
PL: Polysaccharide lyase
SDR: Short-chain dehydrogenases/reductases
SM: Secondary metabolite
TEF1: Translation elongation factor 1-α
TF: Transcription factor
TRI: Trichothecenes biosynthetic cluster
TS: Terpene synthase
