# Supplementary figures and images for: Genomic characterization of Trichoderma atrobrunneum (T. harzianum species complex) ITEM 908: insight into the genetic endowment of a multi-target biocontrol strain
Source: BMC Genomics. 2018 Sep 11;19:662. doi: 10.1186/s12864-018-5049-3 (PMC6131884; doi:10.1186/s12864-018-5049-3)

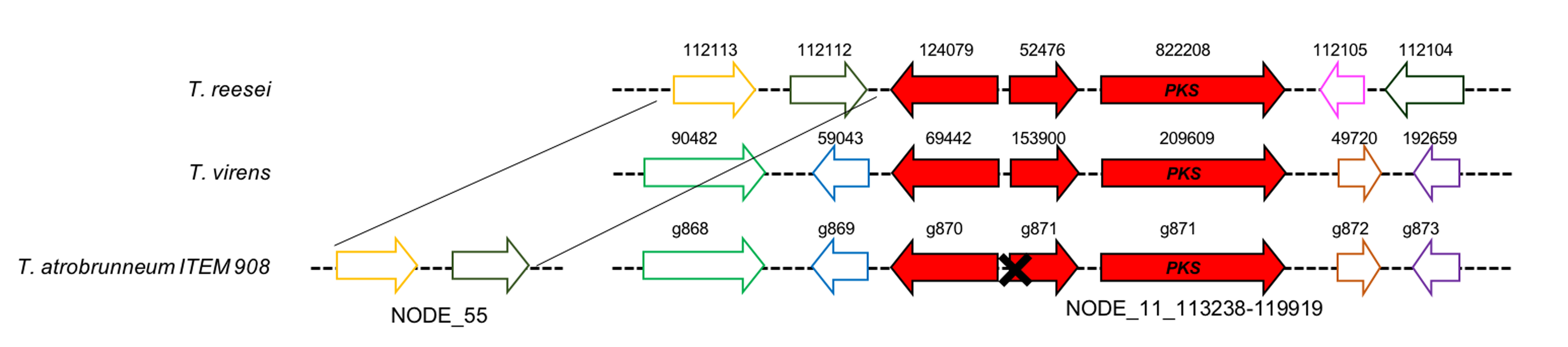

Supplement: Supplementary file 7 — Figure S1. The putative conidial pigment PKS gene clusters of Trichoderma spp. Numbers over the arrows in T. virens and T. reesei indicated the ID of genes as reported in Ensembl Fungi©. Numbers over the arrows in T. atrobrunneum indicated the ID of genes as predicted by Augustus [100]. (PNG 844 kb) [file 12864_2018_5049_MOESM7_ESM.png]

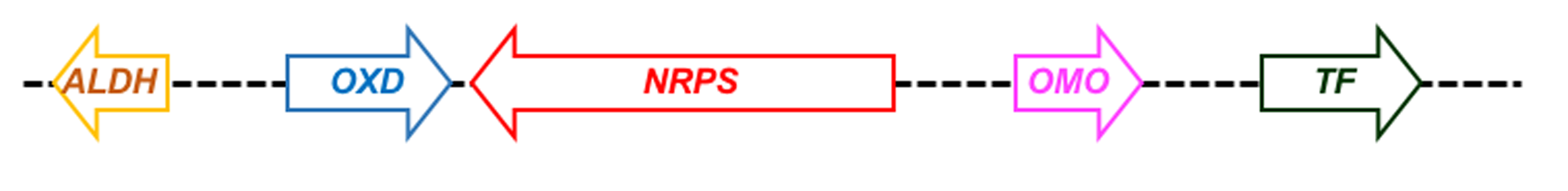

Supplement: Supplementary file 8 — Figure S2. Ferricrocin gene cluster in T. atrobrunneum ITEM 908. ALDH: aldehyde dehydrogenase; OXD: oxidoreductase; NRPS: non-ribosomal peptide synthetase; OMO: ornithine monooxygenase; TF: transcription factor. (PNG 142 kb) [file 12864_2018_5049_MOESM8_ESM.png]
