# Supplementary material for: Genomic characterization of Trichoderma atrobrunneum (T. harzianum species complex) ITEM 908: insight into the genetic endowment of a multi-target biocontrol strain
Source: BMC Genomics. 2018 Sep 11;19:662. doi: 10.1186/s12864-018-5049-3 (PMC6131884; doi:10.1186/s12864-018-5049-3)
Supplement: Supplementary file 9 — Table S3. Trichoderma spp. and genomic sequences accession used in this study. (DOCX 13 kb) [file 12864_2018_5049_MOESM9_ESM.docx]

| **Table S3. *Trichoderma* spp. and genomic sequence accessions used in this study** | | |
| --- | --- | --- |
| **Species** | **Strain** | **Accession** |
| *T. asperellum* | B05 | JNNP01.1 |
| *T. atrobrunneum* | ITEM 908 | PNRQ0000000 |
| *T. atroviride* | IMI 204060 | ABDG02.1 |
| *T. atroviride* | JMC9410 | BCFX01.1 |
| *T. atroviride* | XS2015 | JZUQ01.1 |
| *T. gamsii* | T6085 | JPDN01.1 |
| *T. guizhouense* | NJAU 4742 | LVVK01.1 |
| *T. hamatum* | GD12 | ANCB02.1 |
| *T. harzianum* | B97 | MRYK01.1 |
| *T. harziaunum* | T6776 | JOKZ01.1 |
| *T. koningii* | JMC 1883 | BCGH01.1 |
| *T. longibranchiatum* | SMF2 | ANBJ01.1 |
| *T. parareesei* | CBS 125925 | LFMI01.1 |
| *T. pleuroti* | TPhu1 | MDJU01.1 |
| *T. reesei* | QM6a | AAIL02.1 |
| *T. reesei* | RUT C 30 | JABP01.1 |
| *T. virens* | Gv29-8 | ABDF02.1 |
| *T. virens* | FT 333 | JTGJ01.1 |
| *T. virens* | IMI 304061 | LQCH01.1 |
| *T. virens* | IMV 00454 | MSJK01.1 |
